# Role of Oxidative Stress, Methionine Oxidation and Methionine Sulfoxide Reductases (MSR) in Alzheimer’s Disease

**DOI:** 10.3390/antiox13010021

**Published:** 2023-12-21

**Authors:** Sanjana Chandran, David Binninger

**Affiliations:** 1 Department of Molecular and Integrative Physiology, University of Michigan Medical School, University of Michigan, Ann Arbor, MI 48109, USA; sanjanch@umich.edu; 2 Department of Biological Sciences, Charles E. Schmidt College of Science, Florida Atlantic University, Boca Raton, FL 33431, USA

**Keywords:** Alzheimer’s disease (AD), reactive oxygen species (ROS), methionine (Met), methionine sulfoxide reductase (MSR), oxidative stress, neurodegeneration, amyloid beta (Aβ), tau

## Abstract

A major contributor to dementia seen in aging is Alzheimer’s disease (AD). Amyloid beta (Aβ), a main component of senile plaques (SPs) in AD, induces neuronal death through damage to cellular organelles and structures, caused by oxidation of important molecules such as proteins by reactive oxygen species (ROS). Hyperphosphorylation and accumulation of the protein tau in the microtubules within the brain also promote ROS production. Methionine, a residue of proteins, is particularly sensitive to oxidation by ROS. One of the enzyme systems that reverses the oxidative damage in mammalian cells is the enzyme system known as Methionine Sulfoxide Reductases (MSRs). The components of the MSR system, namely MSRA and MSRB, reduce oxidized forms of methionine (Met-(o)) in proteins back to methionine (Met). Furthermore, the MSRs scavenge ROS by allowing methionine residues in proteins to utilize their antioxidant properties. This review aims to improve the understanding of the role of the MSR system of enzymes in reducing cellular oxidative damage and AD pathogenesis, which may contribute to effective therapeutic approaches for AD by targeting the MSR system.

## 1. Alzheimer’s Disease: Occurrence, Etiology and Pathophysiology

Alzheimer’s disease (AD) is a progressive neurodegenerative disease that disrupts the normal brain structure and function significantly, leading to memory loss. The likelihood of developing AD increases with age, especially after 65 years. Dementia involves the progressive deterioration of memory and intellectual function, and AD is the most common type of dementia [1,2]. Other typical symptoms of AD include spatial awareness impairment, movement dysfunction, depression, delusion, and hallucination. Patients often experience anomic aphasia, acalculia, and apathy. As the disease progresses to the final stages, patients lose their abilities to communicate verbally, to perform activities and functions of daily living and cannot function independently [3,4].

According to the 2019 World Alzheimer Report, the number of people living with dementia already exceeds 50 million people, and this number is expected to more than triple by 2050 as the world population ages and life expectancy continues to increase [5].

Proteinopathies, such as amyloidopathy and tauopathy, oxidative stress, metabolic impairments and higher levels of proinflammatory cytokines such as tumor necrosis factor alpha (TNF-α), interleukin-1β (IL-1β), IL-6, IL-8, and IL-10 in the brain, are associated with AD [6].

Amyloidopathy is the build-up of a peptide named amyloid beta (Aβ) in regions of the brain such as the parenchyma. Aβ is believed to play a key role in the AD etiology [7]. Aβ is produced from amyloid precursor protein (APP), which is present in many tissues of the human body, including in the central nervous system. Under normal non-amyloidogenic conditions, Aβ plays a regulatory role in processes such as axonal growth and influences synaptic plasticity [4,8]. The APP is metabolized through the secretory pathway, which involves transport of APP from the endoplasmic reticulum to the Golgi apparatus and further to the cellular membrane, where it is proteolytically modified. Under non-amyloidogenic conditions, the enzyme α-secretase cleaves APP into two parts: a soluble sAPPα protein and another fragment that is further proteolyzed by the enzyme γ-secretase, resulting in an intracellular APP domain (AICD) and a p3 fragment. However, under amyloidogenic conditions, APP enters a different endosomal–lysosomal pathway and undergoes an alternative biochemical modification, mediated by the β-secretase enzyme (BACE1). BACE1 works near the APP N-terminus, close to the lumen of the cellular organelles. On the other hand, the γ-secretase enzyme operates in close proximity to the C-terminus of APP, which is inside the cytoplasm. The proteolytic modification catalyzed by BACE1 produces an sAPPβ protein and a C99 fragment. The cleavage of the C99 fragment by γ-secretase produces another insoluble form of Aβ, that is released into the extracellular space. The frequency of this pathological APP cleavage by BACE1 is approximately 50% higher in AD patients compared to healthy individuals. The considerably higher amounts of insoluble Aβ in the extracellular space bind to apolipoprotein E (APOE), degenerated axons, microglia, and proinflammatory cytokine-activated astrocytes, resulting in the formation of senile plaques (SPs) [4,8,9,10].

These SPs can enter the blood vessels and reduce the blood supply to the brain. Additionally, they cause damage to neurons and increase neuroinflammation by activating microglia and astrocytes. The higher level of neuroinflammation increases the production of free radicals, such as ROS, and triggers neuronal apoptosis.

Another contributor to neuronal dysfunction and apoptosis is the synthesis of proinflammatory molecules such as prostaglandins, excitotoxins, and cytokines, including tumor necrosis factor α (TNF-α), induced by the stimulation of the receptors for advanced glycation end products on the surface of neurons by Aβ [3,8].

**Figure 1:**
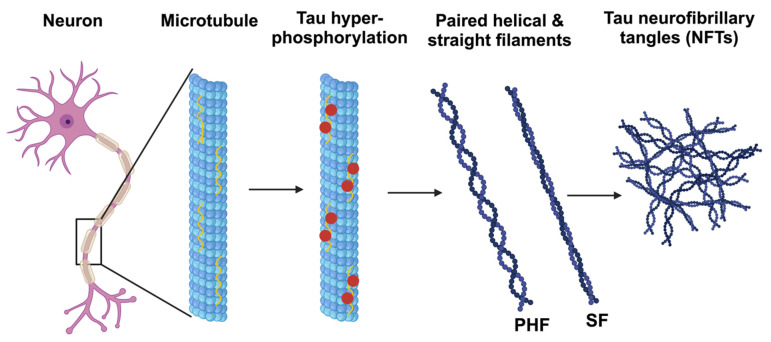
Hyperphosphorylation of tau protein. Hyperphosphorylation of the tau protein causes the microtubules to disassemble into paired helical and straight filaments, resulting in tau neurofibrillary tangles (NFTs). Adapted from [13]. Created with BioRender.com (accessed on 29 August 2023).

Aβ can also induce other pathological changes in neurons which affect their structure, such as the formation of tau neurofibrillary tangles (Figure 1). Microtubules are fragile structural elements of the cytoskeleton that need to be stabilized. They facilitate cellular transport of proteins and other molecules to enable proper functioning of neurons, including appropriate synaptic signaling [3,11,12]. Under physiological conditions, the microtubules are stabilized through the interactions between their basic constituents, tubulins, and tau proteins.

However, due to oxidative stress from the high levels of ROS, tau proteins undergo hyperphosphorylation, which destabilizes the microtubules and induces their disassembly from the cytoskeleton. The disassembly of microtubules induces cytoskeleton deformation, disrupts the intracellular transport and eventually leads to complete neuronal dysfunction. This process promotes the formation of tau tangles, which in turn form toxic aggregates, constituting tauopathy. These aggregates activate the microglia and induce inflammation, leading to neuronal damage and death [11,12]. Tauopathy also contributes to oxidative stress by increasing ROS production.

## 2. Oxidative Stress Due to ROS-Induced Mechanisms

**Figure 2:**
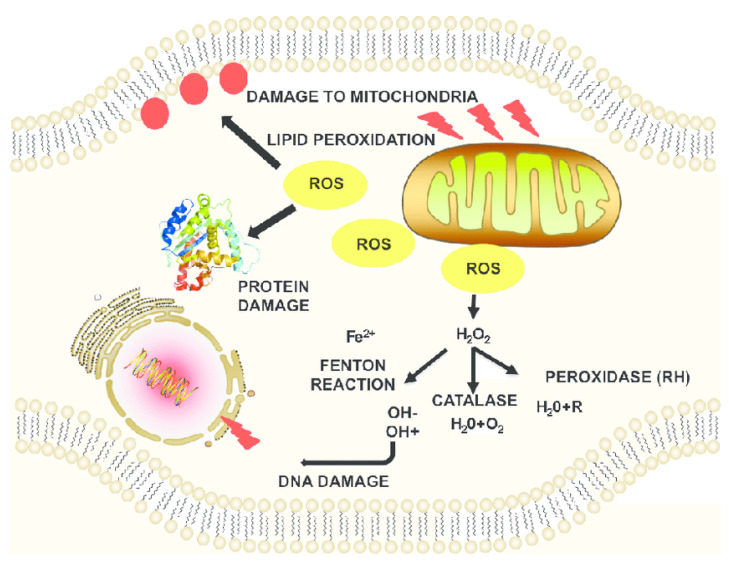
ROS-induced damage to cellular macromolecules and structures. The production of intracellular ROS can damage macromolecules such as DNA, proteins, and lipid bilayers. Adapted from [22].

ROS are mainly produced by mitochondria during normal metabolism in cells. They function as secondary signaling molecules that regulate various biological and physiological processes, including proliferation, host defense, and gene expression, when present in appropriately low amounts [14,15]. However, when ROS levels increase to abnormally high levels, they can damage macromolecules and structures present in cells such as lipids, proteins, and DNA (Figure 2). For example, ROS can react with nucleic acids to induce single- and double-stranded DNA breaks, introducing mutations in the protein-coding region of mitochondrial DNA (mtDNA), and dysregulating the mitochondrial metabolism [16,17]. mtDNA mutations impair the functions of the mitochondrial respiratory chain, diminishing its control over ROS production [14]. The excessive ROS produced can modify DNA repair genes, impairing their ability to identify and repair damaged DNA, which in turn can disrupt cellular processes. These disruptions can further increase ROS production in a vicious cycle, elevating oxidative stress. This elevation in oxidative stress damages mtDNA, inhibits mitochondrial respiratory chain transition, and increases mitochondrial membrane permeability, often triggering cell apoptosis in the absence of sufficient mechanisms to reduce the ROS to an acceptable level [18]. The disruption of the oxidant–antioxidant balance in the brain results in excessively high levels of ROS and free radicals, leading to oxidative stress [19]. Methionine is very important for DNA synthesis, and the increased oxidation of methionine residues due to excessive ROS damages mtDNA. Without ROS reduction mechanisms, the elevated ROS production induces oxidative stress which in turn increases ROS production in a vicious cycle [20]. Higher levels of ROS also have a deleterious effect on structural and enzymatic proteins via oxidation of amino acids, non-amino acid cofactors, formation of cross-links and protein aggregates, and breakdown of proteins [17]. Excessive ROS also increases lipid peroxidation (auto-oxidation). Polyunsaturated fatty acids (PUFAs) are easily oxidized due to several double bonds in their structure, which produces ROS and other reactive organic free radicals [17,18,21].

ROS has also been attributed to the increased production of advanced glycation end products (AGEs). AGEs bind to specific receptors for advanced glycation end products (RAGEs) and disrupt normal cellular functions directly or indirectly through the AGE/RAGE pathway [23]. Increased production of AGEs eventually leads to ROS generation in a cyclical loop.

Oxidative stress increases with physiological aging, and the ability to respond to oxidative stress declines with age. Hence, AGEs accumulate in various tissues with age and alter the function of many proteins, notably those involved in regulating gene transcription [18,22]. As AGEs accumulate in the central nervous system, they act as powerful neurotoxins [24]. The start of protein glycation is a nonenzymatic process with a free amino acid that can produce an unstable Schiff base. The non-enzymatic process occurs along with the unconstrained condensation of aldehyde or ketone groups reportedly present in sugars. These processes follow the Maillard reaction, first reported in 1912 [25,26]. A series of subsequent reactions produce AGEs with irreversibly cross-linked heterogeneous protein aggregates.

Available evidence suggests that AD symptoms manifest only after oxidative stress increases and causes damage seen not only in the core susceptible regions of the brain but also in periphery [25,27,28,29]. Disruption of the proteasomal and/or lysosomal pathway necessary to break down and clear a specific protein or transcriptional activation or rapid translation of an abnormal mRNA can induce the build-up of a specific toxic protein [30,31,32]. Sometimes, a toxic protein is produced by a gene mutation and is unable to be degraded and cleared by the proteasome. Such toxic proteins can then accumulate and form protein aggregates.

## 3. Interplay between Oxidative Stress and Various Molecular Mechanisms of AD

AD brains display significant accumulation of oxidative damage markers of lipid, protein and DNA and transition metals such as Fe, Cu and Zn, as well as impaired antioxidant defense [33]. The recent redox proteomics analysis of the postmortem AD brain has demonstrated oxidative damage to key enzymes involved in energy metabolism, neurotransmission, mitochondrial and proteasomal functions [34]. Oxidative stress can contribute to amyloidopathy by impacting APP gene transcription or APP mRNA translation, proteasomal degradation of APP and Aβ peptides, as well as interactions of APP with transition metals. Transcription factors such as HSF-1 and NF-κB, which are upregulated by ROS, can bind to the promoter sites of the APP gene and induce APP expression [35,36]. The increased APP expression due to NF-κB and ROS has been seen in various experimental models [37,38]. Intracellular Fe plays a key role in the post-transcriptional control of APP expression. The iron-responsive element (IRE) in the APP mRNA has a 5′-untranslated region (UTR) stem–loop arrangement to which the IRE-binding protein (IREBP) binds and downregulates translation. When the intracellular Fe level increases, IREBP dissociates from the binding site at the 5′-UTR of the IRE in the APP mRNA. Akin to the translational regulation of ferritin by iron, the dissociation of IREBP upregulates translation of APP mRNA [39].

Fe is also an important catalyst for ROS production, and elevated levels of Fe have been reported from postmortem analyses of AD brains, suggesting a linkage between Fe, oxidative stress and APP production in the AD brain [40]. ROS responsive transcription factors, such as Sp1, NF-κB, and HIF-1α, can bind to several sites near the promoter region of the BACE1 gene, and hence, ROS levels can modulate BACE1 expression [41,42]. However, the effects of these transcription factors on BACE1 expression are complex. Both upregulation and downregulation of BAC1 expression by the binding of these transcription factors has been reported, as the effects of these transcription factors are sensitive to the cell types and the physiological or experimental conditions of the cells [43]. For example, in the case of NF-κB binding, which NF-κB subunit binds to the sites near the promoter region of the BACE1 gene may determine whether BACE1 expression is upregulated or downregulated [41]. Additionally, although NF-κB is responsive to ROS, its interaction with ROS is complex and certain experimental conditions may favor NF-κB activation and nuclear translocation whereas other conditions may inhibit nuclear translocation of NF-κB [44]. However, higher BACE1 expression due to oxidative stress has been reported in several experimental models [42,45]. Oxidative stress induced by the products of oxidative damage (such as 4-hydroxynonenal [4-HNE]) or oxidizing agents (such as H_2_O_2_ and iron–ascorbate mixture) have been shown to activate specific protein kinases which in turn increase BACE1 activity [46,47]. A more in depth study has shown that increased BACE1 activity, both in vitro and in vivo, due to oxidative stress requires γ-secretase and the JNK/c-jun pathway to be activated [48]. Furthermore, oxidative stress-induced increase in the BACE1 activity at the translational level involving double-stranded RNA-dependent protein kinase (PKR) and eukaryotic initiation factor-2 (eIF2) phosphorylation has been reported [49]. These studies support the role of oxidative stress in increasing BACE1 activity in AD. The release of Aβ42 from APP requires the γ-secretase enzyme complex, composed of presenilin1 (PS1), nicastrin, PEN2 and APH1, and oxidative stress increases the γ-secretase enzyme complex activity by upregulating PS1 [48]. Various studies have shown oxidative stress to enhance the production and accumulation of Aβ42 peptides through its effects on APP metabolism [50].

**Figure 3:**
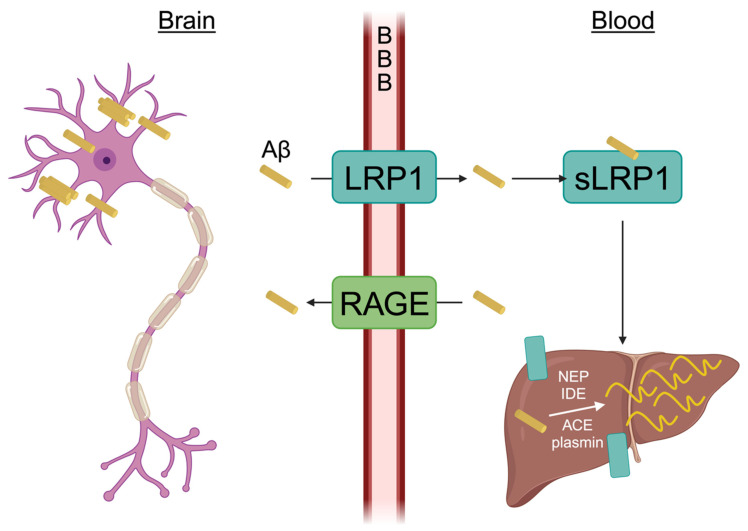
Regulation of clearance of Aβ from the brain. LRP1 shuttles monomeric Aβ through the blood brain barrier (BBB) and into the circulation while RAGE mediates Aβ influx. Through a few Aβ-degrading enzymes, such as NEP, insulin-degrading enzyme (IDE), angiotensin-converting enzyme (ACE), and plasmin, sLRP1 and hepatic LRP1 aid in the degradation of Aβ. Adapted from [54]. Created with BioRender.com (accessed on 29 August 2023).

Not just limiting the production and accumulation of Aβ42 peptides in the brain, their clearance from the brain is also important to ameliorate AD. Oxidative stress-induced damage can impair the clearance of Aβ brain in AD. As shown in Figure 3, the low-density lipoprotein receptor-related protein 1 (LRP1) and the receptor for advanced glycation end products (RAGE) mediate the clearance of Aβ from the brain [51,52,53]. There are two forms of LRP1. One form is the membrane-bound form which is responsible for the transfer of cerebral amyloid beta across the blood–brain barrier (BBB) to the circulating blood and is expressed in neurons, astrocytes, vascular endothelial cells and smooth muscle cells. The other soluble form binds the amyloid beta in the peripheral circulation [51,52]. On the contrary, RAGE, which is present in the BBB, may allow the reentry of amyloid beta from the peripheral circulation into the brain. Under oxidative stress, both forms of LRP1 are altered. The altered form of the membrane-bound LRP1 prevents the clearance of cerebral amyloid beta across the BBB and the oxidized form of the soluble LRP1 does not bind the circulating amyloid beta, allowing its reentry into the brain [53].

### 3.1. Aβ Induced Oxidative Stress

Oxidative damage in the AD brain can be produced by a multitude of mechanisms, but in particular, a large number of in vitro and in vivo studies have pointed to Aβ-induced ROS generation [55,56,57,58,59]. The mechanisms through which Aβ42 and Aβ40 can bind transition metals in a redox-active form through amino acid residues, such as His6, His13 and His14, have been elucidated. The redox-cycling reactions that generate ROS involve the bound transition metal ions and may require Met35 of the Aβ peptide or another reducing agent in the surroundings [60]. Since there is clear postmortem evidence of higher levels of transition metals in the AD brain, especially near the plaque, these Aβ-mediated redox-cycling reactions that generate ROS could be important in AD development. However, other studies propose that Aβ plays an antioxidant and protective role. This involves Aβ scavenging reactive radicals from lipid oxidation, sequestering transition metals to prevent their participation in ROS-producing redox-cycling reactions, and preventing the generation of oxygen free radicals in mitochondria [61]. In addition to the redox-cycling reactions, Aβ may increase intracellular ROS, thus promoting neuronal apoptosis triggered by ROS activation of the apoptosis signal regulating kinase 1 (ASK1) [55]. Another mechanism through which Aβ increases ROS production is through the activation of nicotinamide adenine dinucleotide phosphate (NADPH) oxidase. The higher generation of oxygen radicals in the mitochondria is preventable by antioxidants that target mitochondrial respiration [62,63]. Outside of neurons, Aβ-induced ROS production involves activation of microglia and priming by the soluble or fibrillar form of Aβ, as seen in primary cultures of microglia or coculture of microglia and neurons [64,65]. Through various receptors, Aβ activates microglia that produce both ROS and proinflammatory cytokines such as IL-6, IL-1β, TNF-α, to induce the inflammatory response [66]. For example, fibrillar Aβ acts on a B-type of scavenger receptor in the microglia called CD36 to increase the production of microglial ROS and cytokines and increase phagocytosis [66,67]. Other studies have additionally shown that Aβ-mediated microglial activation and resulting ROS involve the MAC-1 receptor and PI3K, activating NADPH oxidase [64]. The involvement of NADPH oxidase in the Aβ-mediated microglial production of ROS has been shown in several other studies [64,67].

### 3.2. Oxidative Stress and Tau Phosphorylation

Accumulation of hyperphosphorylated tau protein to form neurofibrillary tangles is also seen in AD and enhances the axonal degeneration and synaptic dysfunction that accompany AD [68,69]. Multiple phosphorylation sites in the C-terminal and in the proline-rich regions of the tau protein are phosphorylation targets of kinases such as GSK3β and CDK5. On the contrary, tau can be dephosphorylated by multiple phosphatases, such as PP1, PP2A, and PP2B [69]. Although there is not a complete understanding of the exact mechanisms involved in AD, the activity of GSK3β and CDK5 is enhanced while PP2A activity is diminished, resulting in increased phosphorylation of tau protein. The reduced PP2A activity, along with the activation of JNK and p38 kinase, due to chronic OS in the form of glutathione depletion, has been shown to increase tau phosphorylation in cultured M17 neuroblastoma cells [70]. Another study has shown that Aβ-induced tau phosphorylation in cultured cortical neurons is mediated by OS through the involvement of p38 kinase and the Aβ-induced tau phosphorylation can be prevented by the antioxidant Trolox [71]. Similarly, in rat primary cortical neuronal culture, OS induced by a combination of Fe^2+^ and H_2_O_2_ increases tau phosphorylation, that may be caused by increased activity of GSK3 kinase [72]. On the contrary, there are reports of decreased phosphorylation of tau under OS in different model systems due to the balance of activity of kinases (such as GSK3 or CDK5) and phosphatases (such as PP1) favoring phosphatases. Thus, the link between OS and tau phosphorylation is somewhat nebulous [73,74].

## 4. Role of Methionine and MSR in Aβ Aggregation, Mitochondrial Dysfunction and Neurotoxicity

Many amino acid residues of proteins are susceptible to oxidation by ROS. Methionine is one such residue that is highly susceptible to oxidation in vivo, particularly under conditions of oxidative stress. For example, the sulfoxide form has been found to comprise 10–50% of Aβ in amyloid plaques of AD brain, although it is difficult to determine whether its existence in the plaques contributes to AD etiology or results from the highly oxidative environment around the amyloid plaques, where Aβ may be trapped for long periods [75,76,77,78]. Oxidation of methionine to met-(o) is reversible, and the reverse reaction is catalyzed in vivo by the methionine sulfoxide reductase (MSR) system, comprising peptide-methionine (*S*)-S-oxide reductase (MSRA) and peptide-methionine (*R*)-S-oxide reductase (MSRB), which reduce the *S* and *R* enantiomers, respectively, of the sulfoxide group. Thus, these enzymes provide both an efficient repair mechanism for oxidative damage to methionine residues and general protection against oxidative stress by scavenging reactive oxygen species through the recycling of methionine [79].

Mammalian MSRA is encoded by a single gene and is found in both the cytosol and mitochondria [80,81]. MSRA-knockout (MSRA-KO) mice have been shown to be more vulnerable to the OS and its deleterious effects seen in AD [82,83]. Conversely, overexpression of MSRA in various experimental models has been shown to protect against oxidative stress and improve survival rate [84,85,86]. A cell culture, comprising human neuroblastoma (IMR-32) cells exhibiting AD features, showed elevated MSRA activity and higher MSRA mRNA levels when treated with the sulfoxide form of Aβ42, suggesting that the cells upregulated MSRA to increase cellular protection in the presence of met-(o) in Aβ [87]. Increased total MSR activity, including both MSRA and MSRB, in primary rat hippocampal and cortical neurons, has been shown to prevent the sulfoxide forms of Aβ40 or Aβ42 from inducing cellular apoptosis [88].

Additionally, the absence of MSRA in the cortical neurons of wild-type (WT) and *MSRA*-KO mice to Aβ42 and met-(o)-Aβ negates the neuroprotection provided by met-(o)-Aβ [86]. In another study involving differentiated cells of the rat pheochromocytoma (PC-12), MSRA overexpression significantly increased the resistance of the cells to Aβ42-induced toxicity [89]. These studies indicate that the increased expression of the MSR enzymes confers neuroprotection against oxidative stress in AD. Aging, independent of any disease, induces a decline in in MSR activity based on studies involving WT mice and the decline in MSR activity correlated with reduced MSRA mRNA levels [89,90,91,92]. Overall, the aggregate data from various human and animal studies suggest that the low MSR levels observed in AD brain can be attributed to aging-induced lower transcription, as well as disease-induced defects in translational or post-translational mechanisms. The oxidation of the sole methionine residue of proteins such as Aβ to form protein-met-(o) disrupts the fibrillar structure and makes it easier for the protein-met-(o) to accumulate [93]. The decline in MSR activity facilitates a build-up of protein-met-(o) [94]. Thus, both reduced MSR activity and the build-up of Aβ-met-(o) increase the levels of soluble Aβ oligomers, which disrupt mitochondrial function.

Since soluble Aβ oligomers are believed to be more neurotoxic than only Aβ aggregates, pharmacologically upregulating MSRA transcription to increase its activity may have therapeutic benefits [95,96]. Various compounds such as pergolide, pergolide sulfoxide, and *S*-adenosyl methionine have been shown to upregulate MSRA transcription and activity in neurons [97]. APP^+^ type of mice, immunized with methionine sulfoxide-rich protein, exhibited a lower amyloid plaque burden in the hippocampus, presumably by reducing the build-up of protein-met-(o) (including Aβ-met-(o)) in the brain [88]. Peripheral administration of anti-Aβ antibody fragments encapsulated in polymeric nanomicelles has also been shown to reduce various toxic Aβ species in the brain [98].

The presence of MSR enzymes in the mitochondria is an important facet of its role in cellular protection against oxidative stress in AD. Numerous studies focused on the role of mitochondria in normal aging and neurodegenerative diseases have established the link between mitochondrial dysfunction and AD. For example, platelets and brains of AD patients and others exhibiting mild cognitive dysfunction show reduced cytochrome c oxidase (mitochondrial respiratory Complex IV) activity, presumably due to aging-driven increase in mitochondrial oxidative stress [99,100]. MSRA deficiency increases mitochondrial dysfunction in APP^+^ mice. The aging-driven reduction in MSRA expression can combine with elevated Aβ levels to exacerbate mitochondrial dysfunction, leading to AD [90,91,92].

MSRB overexpression in APP/PS1 mice has also been shown to reduce Aβ production by reducing APP and BACE1 expression but not the expression of γ-secretase and ADAM10 [101]. Higher levels of MSRB also have been shown to reduce the activity of JNK through phosphorylation, thus limiting neuroinflammation and associated neuronal apoptosis [96]. Finally, MSRB also has a beneficial effect of reducing risk of apoptosis by downregulating the expression of pro-apoptosis proteins, such as Bax and caspase3, and upregulating the expression of anti-apoptotic protein Bcl2 [101].

## 5. Role of Methionine and MSR in the Intracellular Calcium (Ca^2+^) Homeostasis

There is significant evidence that intracellular calcium (Ca^2+^) homeostasis is disrupted in both sporadic and familial forms of AD and can exacerbate Aβ formation and promote tau hyperphosphorylation [102,103]. Additionally, Aβ can influence cellular pathways involved in Ca^2+^ buffering, compromising the ability of neurons to respond to excitotoxic challenge [104]. This could be indicative of a pathogenic feed-forward cycle where Aβ and Ca^2+^ can concomitantly impair synaptic morphology, trigger neuronal apoptosis, and eventually lead to deterioration of cognition [105]. The mediators of the pathogenic feed-forward cycle presumably lie downstream of the Ca^2+^ signaling and are also present in excitatory synapses where Aβ oligomers likely initially bind. One such mediator is a post-synaptic protein present in excitatory synapses called the Ca^2+^/calmodulin (CaM)-dependent protein kinase II (CaMKII). A progressive increase in the oxidation of Met residues in calmodulin in the rat brain has been attributed to the protein losing its ability to regulate plasma membrane ATP hydrolysis and ATP-dependent Ca^2+^ transport [106,107]. Other studies have shown that the exposure of calmodulin to ROS resulted in oxidation of methionine to methionine sulfoxide whereas protein-bound methionine sulfoxide in calmodulin is reduced by MSR [108,109].

Since APP has been shown to be phosphorylated in neuronal cultures by several kinases including CaMKII, CaMKII may play a key role in Aβ production [110]. The link between CaMKII and Aβ production is further supported by the findings of co-localization of αCaMKII with SPs, with differences in the deposition pattern around diffuse and neuritic plaques [111,112]. Although phosphorylation of CaMKII sites (T654/S655) can change the conformation of APP and regulate its trafficking, there is no direct evidence that CaMKII is involved in APP processing [113,114].

The evidence, however, supports tau phosphorylation by CaMKII. Increased αCaMKII expression in CA1 neurons and increased αCaMKII autophosphorylation in cell bodies of CA3 neurons and granule cells in the DG are clear evidence that αCaMKII is hyperactive in those areas of the brain [111,112,115,116]. This αCaMKII hyperactivity could contribute to hyperphosphorylation of tau at many sites, increasing NFT formation [117]. In AD brains, αCaMKII in cell bodies frequently co-localizes with NFTs or phosphorylated tau [111,112,117,118,119,120,121]. Tau phosphorylation by CaMKII partially inhibits its binding to microtubules, and thus, excessive CaMKII activation can cause hyperphosphorylated tau to form paired helical and straight filaments which then form NFTs, exacerbating AD [122].

The oxidation of methionine residues M281 and M282 activates CaMKII, a process that is reversed by MSRA. Mice that lack MSRA show excessive oxidative activation of CaMKII [123]. This hyperactivation of CaMKII could increase NFT formation and the resulting AD pathogenesis.

## 6. Role of Methionine and MSR on Apolipoprotein A-I (apoA-I) Levels and Its Protective Mechanism

Considerable evidence suggests a protective role of high-density lipoprotein (HDL) and its major apolipoprotein apoA-I, in AD. ApoA-I binds Aβ and reduces Aβ levels in the brain of AD animal models by increasing Aβ clearance from the brain [123]. APP/PS1 mice, which are deficient in apoA-I, have higher Aβ deposition in cerebral blood vessels [124]. Conversely, increasing HDL/apoA-I levels in Aβ overexpressing mice, resulted in reduced Aβ deposition in cerebral blood vessels, lower amyloid plaque load, and attenuated neuroinflammation [125,126].

Oxidation of the methionine residues of apoA-I leads to aggregation of the Met-(o)-apoA-I into fibrils and thus reduces the levels of apoA-I and its protective effect. MSRA overexpression has been found to increase apoA-I levels [127,128].

## 7. Role of Methionine 35 (Met35) Residue of Aβ42 in the Neurotoxicity of the AD Brain

**Figure 4:**
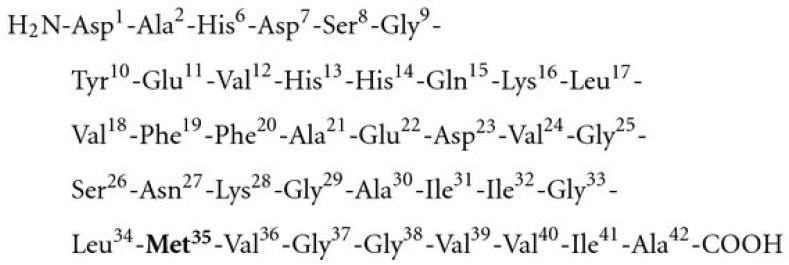
Location of methionine residue in full length amyloid beta peptide. Methionine is located at the 35th position of the amyloid beta peptide. Adapted from [129].

As shown in Figure 4, Aβ42 has methionine present at the 35th residue in the peptide [129]. In the AD brain, a significant fraction of SP-resident Aβ42 has methionine in the form of met-(o) [78]. It has been found that lipid peroxidation initiated by oxidation of the Met35 is an early event in Aβ42 neurotoxicity [130]. The hydrophobic surroundings around Met35 in Aβ42 are important for the oxidative, neurotoxic, and aggregation properties of Aβ42.

In vitro studies have shown that Met35(O)-Aβ42, formed when Met35 is oxidized to the sulfoxide in Aβ42, is toxic compared to WT-Aβ42 and contributes to the increase of soluble Aβ seen in the brain in AD [131]. Furthermore, there is a decrease in the level of MSRA, possibly increasing Met35-(o)-Aβ42 formation [93]. Moreover, substitution of Met35 by norleucine (Nle) was reported to reduce Aβ42 toxicity, further reinforcing the importance of Met35 oxidation to Aβ42 toxicity [132].

Although the Met35 residue in Aβ42 is readily oxidized, the Met35 oxidation only slightly inhibits Aβ fibrillization. Hence, the Met35 oxidation state alone may not have a large effect on the plaque formation in vivo [133]. Thus, the dominant mechanism of Met35(O)-Aβ42 toxicity may be through the elevated levels of soluble Aβ produced in the brain as opposed to Aβ fibrillization. Additional in vivo studies are necessary to completely understand the link between Met35-(o)-Aβ42 and Aβ fibrillization and the effect of the Msr system on the soluble Aβ levels and Aβ fibrillization in the brain.

## 8. Conclusions and Future Perspectives

The oxidation of methionine in proteins present in the brain due to oxidative stress can have detrimental effects on protein stability and function, increasing the risk of AD. While precise data are not available to compare the levels of methionine oxidation in these brain proteins, it has been suggested that the proteins with slow turnover rates are the most likely to have higher levels of methionine oxidation [134,135,136,137,138]. Oxidative damage in the AD brain, particularly due to oxidation of methionine residues in brain proteins, contributes to the disease’s progression by increasing Aβ deposition, tau hyperphosphorylation, neuroinflammation, and neuronal apoptosis.

Although several in vitro and in vivo studies have studied the effects of oxidation of Met35 residue of Aβ42 on Aβ toxicity and its contribution to AD pathology, the precise mechanisms are still not clear and additional in vivo studies are necessary.

The MSR system of enzymes, consisting of MSRA and MSRB, directly or indirectly mitigates AD pathogenesis by reversing the oxidation of the methionine residues. While levels of MSRA and MSRB proteins are reduced in the aging brain, they may be reduced by varying degrees in different regions of the brain, for example, hippocampus versus cortex, and the reduction in MSRA and MSRB protein levels may occur at different times in the different regions of the aging brain. The effects on MSRA and MSRB deficiency in regulating AD pathology may also be different in different regions of the brain. While many studies have focused on the role of MSRA in regulating AD pathology, there have been far fewer studies conducted on the effect of MSRB protein levels on APP processing, Aβ production and tau phosphorylation. Additional studies need to be conducted to completely understand the role of MSRB in regulating AD pathology. In summary, the MSR system of enzymes could be a potential therapeutic target for ameliorating AD pathology and slowing the onset of AD

The MSR system of enzymes is a potential therapeutic target for ameliorating AD pathology and slowing the onset of AD. While pharmacological upregulation of MSRA transcription and activity in neurons has been shown, additional studies are necessary to determine if these compounds can reduce toxic Aβ in the brain and ameliorate AD pathology. Compounds that upregulate MSRB transcription and activity in neurons need to be identified and their effects on toxic Aβ accumulation in the brain need to be investigated. Additionally, what effects, if any, would the compounds that upregulate MSRA and/or MSRB transcription and activity have on tau phosphorylation and NFT formation? This question needs to be answered through additional studies.

## Data Availability

The data supporting the findings of this study are available within the article.
